# Gestational diabetes mellitus aggravates adverse perinatal outcomes in women with intrahepatic cholestasis of pregnancy

**DOI:** 10.1186/s13098-024-01294-z

**Published:** 2024-03-01

**Authors:** Xia Li, Qin-Yu Cai, Xin Luo, Yong-Heng Wang, Li-Zhen Shao, Shu-Juan Luo, Lan Wang, Ying-Xiong Wang, Xia Lan, Tai-Hang Liu

**Affiliations:** 1 Department of Bioinformatics, School of Basic Medical Sciences , Chongqing Medical University, No.1 Yixueyuan Rd, Yuzhong District, 400016 Chongqing, China; 2 Joint International Research Laboratory of Reproduction & Development, Chongqing Medical University, 400016 Chongqing, China; 3 Department of Obstetrics, Women and Children’s Hospital of Chongqing Medical University, 401147 Chongqing, China; 4 Department of Obstetrics, The First Affiliated Hospital of Chongqing Medical University, 400016 Chongqing, China

**Keywords:** Intrahepatic cholestasis of pregnancy, Gestational diabetes mellitus, Perinatal outcome, Bile acid, Preterm labour, Nomogram

## Abstract

**Purpose:**

To evaluate the effect of intrahepatic cholestasis of pregnancy (ICP) with gestational diabetes mellitus (GDM) on perinatal outcomes and establish a prediction model of adverse perinatal outcomes in women with ICP.

**Methods:**

This multicenter retrospective cohort study included the clinical data of 2,178 pregnant women with ICP, including 1,788 women with ICP and 390 co-occurrence ICP and GDM. The data of all subjects were collected from hospital electronic medical records. Univariate and multivariate logistic regression analysis were used to compare the incidence of perinatal outcomes between ICP with GDM group and ICP alone group.

**Results:**

Baseline characteristics of the population revealed that maternal age (*p* < 0.001), pregestational weight (*p* = 0.01), pre-pregnancy BMI (*p* < 0.001), gestational weight gain (*p* < 0.001), assisted reproductive technology (ART) (*p* < 0.001), and total bile acid concentration (*p* = 0.024) may be risk factors for ICP with GDM. Furthermore, ICP with GDM demonstrated a higher association with both polyhydramnios (OR 2.66) and preterm labor (OR 1.67) compared to ICP alone. Further subgroup analysis based on the severity of ICP showed that elevated total bile acid concentrations were closely associated with an increased risk of preterm labour, meconium-stained amniotic fluid, and low birth weight in both ICP alone and ICP with GDM groups. ICP with GDM further worsened these outcomes, especially in women with severe ICP. The nomogram prediction model effectively predicted the occurrence of preterm labour in the ICP population.

**Conclusions:**

ICP with GDM may result in more adverse pregnancy outcomes, which are associated with bile acid concentrations.

**Supplementary Information:**

The online version contains supplementary material available at 10.1186/s13098-024-01294-z.

## Introduction

Intrahepatic cholestasis of pregnancy (ICP) is the most common pregnancy-specific liver disease, and it usually presents in the third trimester [1]. It is characterized by gestational pruritus and elevated serum total bile acid (TBA) concentrations in women. Maternal symptoms and biochemical abnormalities usually subside after delivery. The increase of maternal TBA is the most important laboratory index for the diagnosis of ICP. Elevated TBA concentrations are associated with significant fetal risks, including adverse perinatal outcomes such as preterm labour, meconium-stained amniotic fluid, respiratory distress syndrome, and stillbirth [2–4]. A large Swedish cohort showed that when maternal serum bile acid concentration ≥ 40 µmol/L, the likelihood of spontaneous preterm labour, meconium-stained amniotic fluid, and fetal asphyxia increased significantly [5]. Gestational diabetes mellitus (GDM) is a common pregnancy complication characterized by glucose intolerance of varying severity that occurs or is first discovered during pregnancy [6, 7]. GDM may be explained for the increased risk of pregnancy complications such as preeclampsia, preterm labour, and excessive growth of the fetus [3, 8]. Previous studies have found that pregnant women with ICP were more likely to be with GDM [9]. This is explained by the fact that high bile acid affects gluconeogenesis, insulin secretion, insulin sensitivity, and glycogen synthesis [10–12]. Abnormal bile acid receptor farnesoid X receptor (FXR) in ICP cases also affect glucose metabolism and attenuate gluconeogenesis [13]. However, whether the co-occurrence of ICP and GDM leads to more serious adverse pregnancy outcomes than ICP alone has not been clearly reported. Considering the potential relationship between ICP and GDM, it is important to understand the differences in perinatal outcomes between those with ICP and GDM and those with ICP alone. This understanding is helpful in promoting the development of diagnosis and treatment measures for this population. To investigate this, we conducted a multi-center retrospective cohort study to explore the effects of different TBA concentrations on the incidence of GDM and perinatal outcomes and further to determine whether combined GDM worsened the outcomes. Finally, we developed a predictive model for preterm labour in the ICP population to help clinicians identify the likelihood of preterm labour early and take appropriate intervention measures in a timely manner.

## Methods

### Study participants

This is a multi-center retrospective study conducted in two Grade III and Grade A hospitals in Chongqing, China, including the First Affiliated Hospital of Chongqing Medical University and the Women and Children’s Hospital of Chongqing Medical University. The total number of newborns in the two hospitals exceeded 10,000 and 15,000, respectively, and they are also the two largest maternity hospitals in Chongqing.

Patients diagnosed with ICP during pregnancy from January 2018 to December 2021 in these two hospitals were included in this study. Each pregnant woman underwent routine testing for parameters such as blood routine examination, urine, liver function, kidney function, thyroid function, and TBA following admission. The electronic health records of all included pregnant women were accessed to extract their relevant general clinical data, laboratory biochemistry, and perinatal outcome information. The diagnosis of all ICP cases in this study was confirmed in accordance with the 10th edition of the International Classification of Diseases (ICD-10), relying on the presence of pruritus and bile acids ≥ 10 µmol/L documented in each patient’s record. All subjects underwent 75 g oral glucose tolerance test (OGTT) and venous blood glucose was measured in routine prenatal examination before 24 weeks. GDM was diagnosed from venous samples according to IADPSG / WHO 2010 criteria (fasting blood glucose ≥ 5.1 mmol / L, 1 h blood glucose ≥ 10.0 mmol / L or 2 h blood glucose ≥ 8.5 mmol / L). Women with liver disease or abnormal liver function before pregnancy and women with missing data were excluded. This study was approved by the ethics committee of Chongqing Medical University (ID:2022-011-01 ).

### Data collection and grouping scheme

All data were collected from hospital electronic medical records, including demographic characteristics, pregnancy history, and biochemical indicators. The perinatal outcome indicators included in the study include preeclampsia, anemia during pregnancy, nuchal cord, preterm prelabour rupture of membranes (PPROM), placenta accreta, abnormal placental shape, meconium-stained amniotic fluid, polyhydramnios, oligohydramnios, spontaneous preterm labour, fetal respiratory distress syndrome, fetal macrosomia, fetal growth restriction, admission care, fetal anomaly, fetal chromosomal abnormalities, and low birth weight. In order to protect the privacy of patients, the personal identification information of all cases was deleted in the process of data collection and analysis.

This study established an ICP with GDM group consisting of women with ICP with GDM (*n* = 390) and an ICP group consisting of women with ICP alone (*n* = 1,788). To explore the effect of TBA concentration on perinatal outcome, we categorized three ICP subgroups according to the TBA concentrations, including the mild group (10 ≤ TBA < 40 µmol/L), the moderate group (40 ≤ TBA < 100 µmol/L), and the severe group (TBA ≥ 100 µmol/L). In addition, the women with ICP with GDM were divided into an ursodeoxycholic acid treatment group and a non-treatment group to explore whether drug treatment can save the impact of GDM.

### Construction of nomogram prediction model

All ICP population data included in the study were randomly divided into a training set and validation set according to 4: 1. Based on the training set data, univariate and multivariate regression were used to screen out independent risk factors associated with preterm labour. Univariate logistic regression was used to screen out independent risk factors associated with preterm labour. Multivariable regression analysis is employed to adjust for known confounding factors associated with preterm labour. Then, according to the results of regression analysis, a nomogram was drawn to predict the probability of preterm labour in the ICP population. The area under the curve (AUC) of the receiver operating characteristic (ROC) curve was used to evaluate the predictive ability of the model. A calibration plot was drawn to evaluate the accuracy of the prediction model, and a clinical decision curve was drawn to evaluate the patient benefit of the model. The model was externally validated using the validation set, and the model was evaluated by plotting the ROC curve, calibration plot, and clinical decision curve analysis (DCA).

### Statistical analysis

All data were statistically analyzed using SPSS 24.0 for windows or R.4.2.1. *p* < 0. 05 was considered statistically significant. Continuous variables were tested for normality using the Shapiro-Wilk test. Continuous variables conforming to normal distribution were expressed as mean ± SD and analyzed by independent sample t-test or ANOVA test. Continuous variables with non-normal distribution are expressed as median and quartile ranges, which are compared by Mann-Whitney U or Kruskal-Wallis tests. The categorical variables were described by the number of cases and composition ratio and compared by chi-square or Fisher exact test. Univariate and multivariate logistic regression tests were used to determine the incidence of perinatal outcomes between the ICP with GDM group and the ICP group. The differences in baseline demographic (maternal age, pregestational weight, gestational weight gain, pre-pregnancy body mass index, bile acid concentration, use of ursodeoxycholic acid, assisted reproductive technology (ART), and TBA as confounders were adjusted. We also compared perinatal outcomes between the ICP with GDM group and the ICP group with different ICP severity grades, and further logistic regression analysis was performed on the different outcome variables. A *p* value of < 0.05 and 95% confidence interval (CI) not crossing 1.00 were considered statistically significant.

## Results

### Analysis of population baseline characteristics in women with ICP

**Fig. 1:**
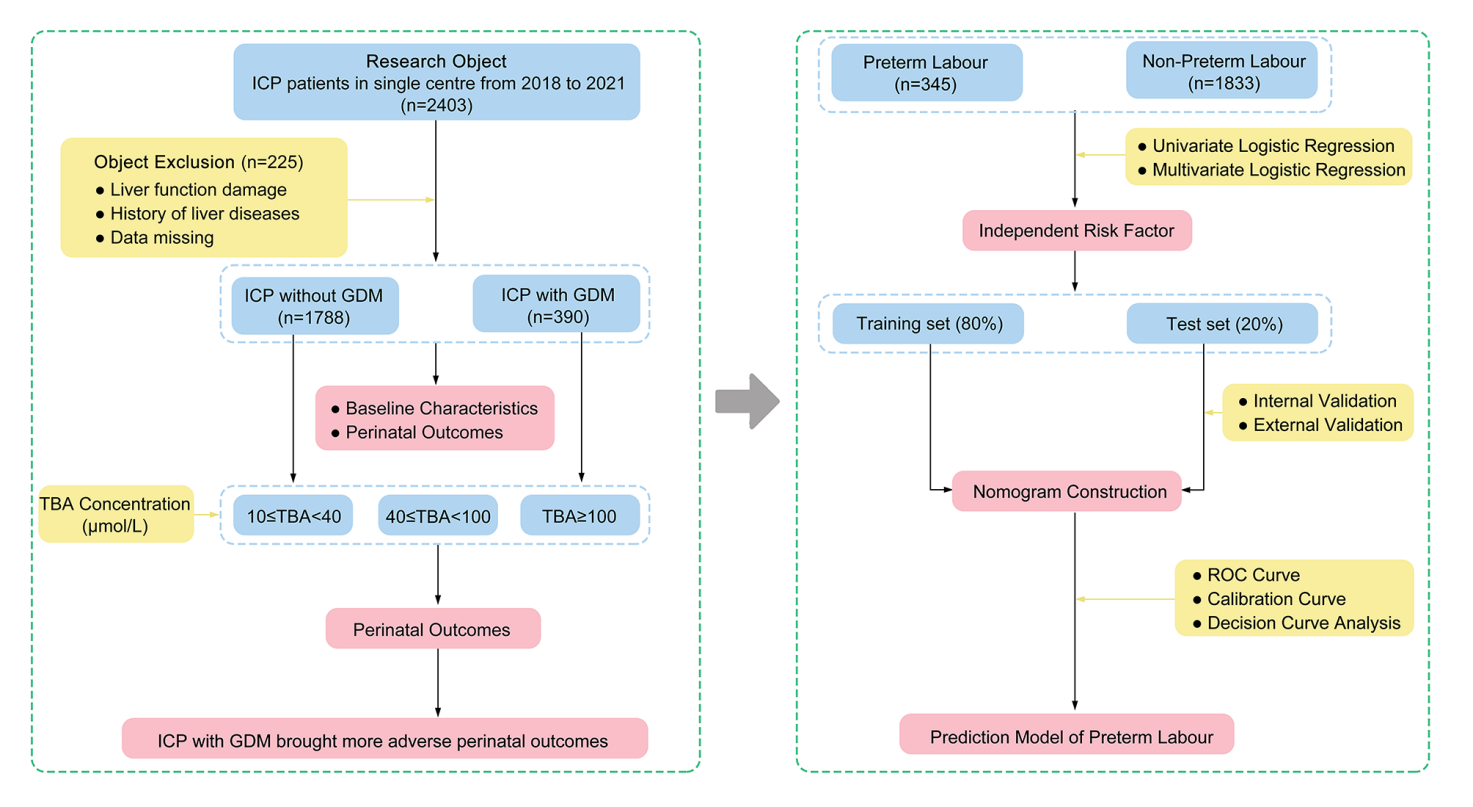
Flowchart showed the study grouping scheme and the analysis process

The 2,178 pregnant women included in this study were divided into two groups, including 1,788 in ICP without GDM group (ICP group) and 390 in ICP with GDM group (Fig. 1). Firstly, the population baseline characteristics of the two groups were analyzed, and the results showed that the maternal age (*p* < 0.001), pregestational weight (*p* = 0.01), pre-pregnancy BMI (*p* < 0.001), gestational weight gain (*p* < 0.001), and assisted reproductive technology (ART) (*p* < 0.001) were the risk factors for ICP with GDM (Table 1). Notably, women in the ICP with GDM group had higher total bile acid (TBA) concentrations than women in the ICP group (20.20 relative to 18.60; *p* = 0.024). Therefore, they were also more likely to use ursodeoxycholic acid after diagnosis (34.4% relative to 40%, *p* = 0.037). In addition, there was no difference in pre-delivery weight, pregnancy BMI, history of ICP, smoking, drinking, and adverse pregnancy history between the ICP group and the ICP with GDM group.

**Table 1:** Baseline characteristics of women with ICP.

Characteristic	ICP with GDM (*n* = 390)	ICP without GDM (*n* = 1,788)	***p***.value
Maternal age, y, mean ± SD	30.99 ± 3.93	29.99 ± 4.28	< 0.001*
Pregestational weight, kg, mean ± SD	53.61 ± 7.23	52.57 ± 7.19	0.010*
Pre-delivery weight, kg, mean ± SD	65.75 ± 7.86	66.46 ± 8.28	0.122
Gestational weight gain, kg, mean ± SD	12.14 ± 4.66	13.88 ± 4.77	< 0.001*
Pre-pregnancy BMI, kg/m^2^, mean ± SD	21.55 ± 2.82	20.91 ± 2.71	< 0.001*
pregnancy BMI, kg/m^2^, mean ± SD	26.42 ± 2.95	26.43 ± 3.10	0.924
History of ICP, n (%)	18(4.6%)	82(4.6%)	0.980
Smoking, n (%)	2(0.5%)	18(1.0%)	0.558
Drinking, n (%)	1(0.3%)	12(0.7%)	0.485
Use of ursodeoxycholic acid, n (%)	134(34.4%)	716(40.0%)	0.037*
Adverse pregnancy history, n (%)	37(9.5%)	133(7.4%)	0.172
ART, n (%)	82(21.0%)	219(12.2%)	< 0.001*
Total bile acid, µmol/L, median (interquartile range)	20.20(13.68, 35.48)	18.60(13.00, 31.70)	0.024*

**Abbreviations**: SD: standard deviation; BMl: body mass index; ICP: intrahepatic cholestasis of pregnancy; ART: assisted reproductive technology

*Significant difference between ICP with GDM group and ICP group

### ICP with GDM will lead to more adverse perinatal outcomes compared to ICP alone

To confirm whether ICP with GDM will lead to more serious adverse consequences, we analyzed the perinatal outcomes in many aspects between the two groups. Univariate analysis showed that the amniotic fluid content of the ICP with GDM group was significantly higher than the ICP group (Table 2). After adjusting for potential confounders, the risk of polyhydramnios increased (4.6% relative to 2.0%; OR 2.66; 95% CI 1.46, 4.86), the risk of oligohydramnios was reduced (5.1% relative to 10.1%; OR 0.52; 9% CI 0.32, 0.83). Moreover, the preterm labour (24.6% relative to 13.9%; OR 1.67; 95% CI 1.26, 2.22) was more likely to occur in the ICP with GDM group after adjusting for potential confounders. Women in ICP with GDM group have a higher proportion of neonatal intensive care unit (NICU) admission (16.9% relative to 11.7%) and low birth weight (26.9% relative to 19.2%); however, the difference was not statistically significant after controlling for confounders. In addition, other indicators, including preeclampsia, anemia during pregnancy, obstetric vaginal laceration, nuchal cord, placental abruption, placenta accreta, abnormal placental shape, fetal respiratory distress syndrome, fetal macrosomia, fetal growth restriction, fetal anomaly, and fetal chromosomal abnormalities, have no significant differences between two groups. ICP with GDM group was further divided into the ursodeoxycholic acid treatment subgroup and non-ursodeoxycholic acid treatment subgroup to confirm whether ursodeoxycholic acid could improve the adverse perinatal outcomes caused by ICP with GDM (Supplementary Table 1). There was no significant difference in perinatal outcomes between the treatment group and the non-treatment group.

**Table 2:** Perinatal outcomes of women with ICP.

Variables	ICP with GDM (*n* = 390)	ICP without GDM (*n* = 1,788)	Univariate analysis		Multivariate analysis*	
			OR (95% CI)	***p***.value	OR(95% CI)	***p***.value
Preeclampsia, n (%)	21(5.4%)	110(6.2%)	0.87(0.54, 1.40)	0.564	0.80(0.48, 1.31)	0.369
Anemia during pregnancy, n (%)	36(9.2%)	205(11.5%)	0.79(0.54, 1.14)	0.203	0.77(0.53, 1.13)	0.186
Nuchal cord, n (%)	111(28.5%)	502(28.1%)	1.02(0.80, 1.30)	0.878	1.07(0.83, 1.37)	0.620
PPROM, n (%)	64(16.4%)	277(15.5%)	1.07(0.80, 1.44)	0.651	1.13(0.83, 1.54)	0.439
Placenta accreta, n (%)	38(9.7%)	130(7.3%)	1.38(0.94, 2.01)	0.098	1.25(0.85, 1.85)	0.257
Abnormal placental shape, n (%)	13(3.3%)	49(13.9%)	1.22(0.66, 2.28)	0.524	1.10(0.58, 2.09)	0.772
Preterm labour, n (%)	96(24.6%)	249(13.9%)	2.02(1.55, 2.63)	< 0.001	1.67(1.26, 2.22)	< 0.001
Fetal respiratory distress syndrome, n (%)	33(8.5%)	140(7.8%)	1.09(0.73, 1.62)	0.676	0.98(0.65, 1.48)	0.940
Fetal macrosomia, n (%)	9(2.3%)	48(2.7%)	0.86(0.42, 1.76)	0.673	1.04(0.50, 2.18)	0.922
Fetal growth restriction, n (%)	8(2.1%)	32(1.8%)	1.15(0.53, 2.51)	0.728	0.94(0.42, 2.11)	0.880
NICU admission, n (%)	66(16.9%)	210(11.7%)	1.53(1.13, 2.07)	0.006	1.28(0.93, 1.76)	0.135
Meconium-stained amniotic fluid, n (%)	59(15.1%)	253(14.1%)	1.08(0.80, 1.47)	0.617	1.01(0.74, 1.39)	0.928
polyhydramnios, n (%)	18(4.6%)	36(2.0%)	2.36(1.32, 4.19)	0.004	2.66(1.46, 4.86)	0.001
Oligohydramnios, n (%)	21(5.4%)	180(10.1%)	0.51(0.32, 0.81)	0.004	0.52(0.32, 0.83)	0.006
Fetal anomaly, n (%)	5(1.3%)	31(1.7%)	0.74(0.28, 1.91)	0.528	0.64(0.24, 1.70)	0.370
Fetal chromosomal abnormalities, n (%)	1(0.3%)	12(0.7%)	0.38(0.05, 2.94)	0.354	0.32(0.40, 2.53)	0.280
Low birth weight, n (%)	105(26.9%)	344(19.2%)	1.55(1.20, 1.99)	0.001	1.21(0.92, 1.60)	0.169

**Abbreviations:** CI: confidence interval; PPROM: preterm prelabor rupture of membranes; NICU: neonatal intensive care unit

* **excluded confounders:** maternal age, pregestational weight, gestational weight gain, pre-pregnancy body mass index, bile acid concentration, use of ursodeoxycholic acid, ART, and total bile acid

### ICP with GDM further aggravates the high incidence of adverse perinatal outcomes in women with severe ICP

**Fig. 2:**
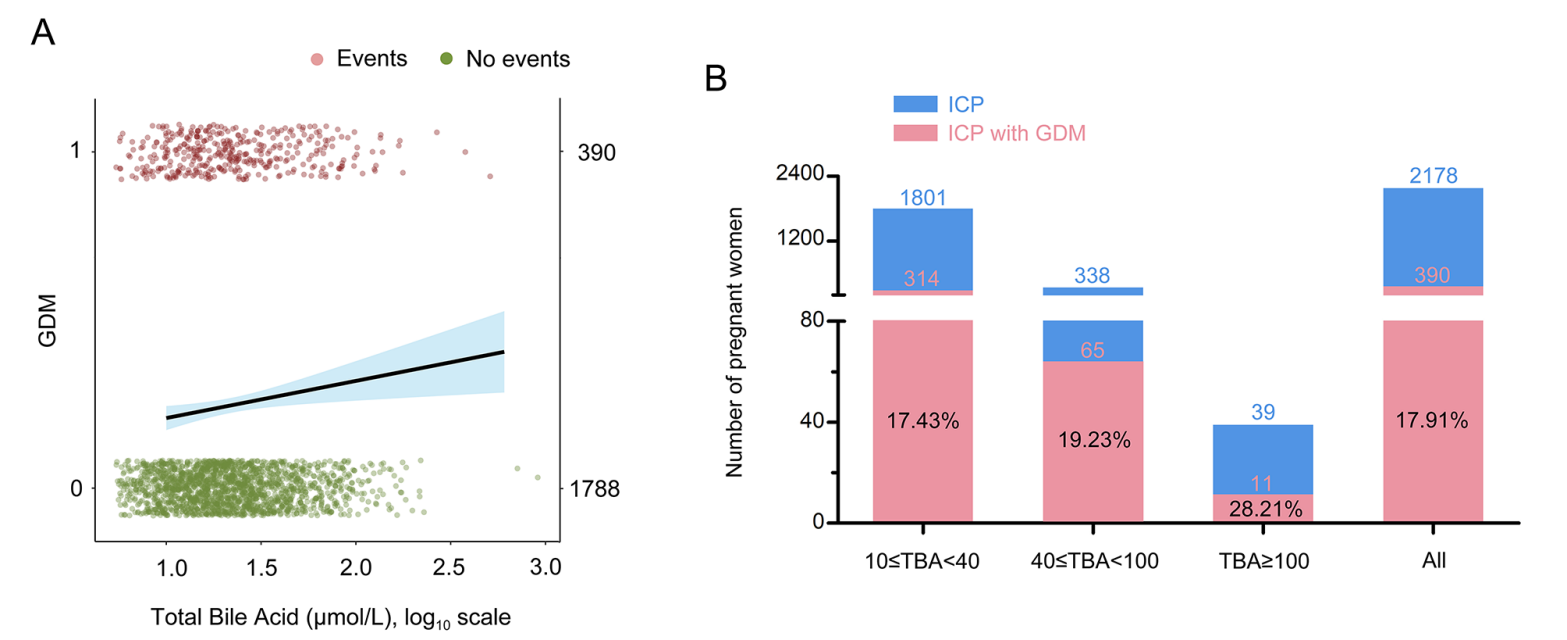
Elevated TBA concentration was closely associated with the increased risk of GDM in women with ICP. **(A)** Simple logistic regression curves showed estimated probabilities and 95% CIs for the association of GDM with maternal serum TBA concentrations. **(B)** Percentage of ICP with GDM population and ICP alone population under different TBA concentrations

**Fig. 3:**
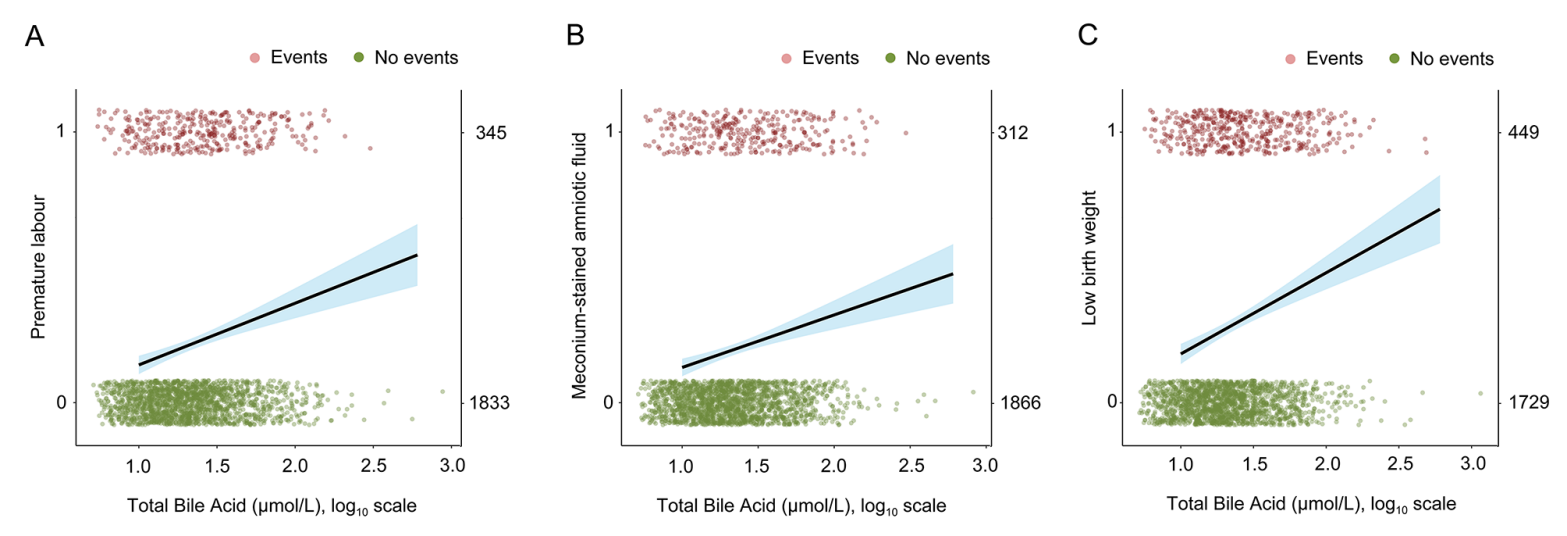
Elevated TBA concentration was closely associated with the increased risk of adverse perinatal outcomes in women with ICP. The simple logistic regression curve showed the estimated probability and 95% CIs of preterm labour **(A)**, meconium-stained amniotic fluid **(B)**, and low birth weight **(C)** in relation to maternal serum TBA concentrations

TBA concentrations determine the severity of ICP, and to investigate the effect of different bile acid concentrations on perinatal outcome, further subgroup analyses were performed. Logistic regression showed that for every doubling of bile acid concentrations, the risk of GDM in the ICP population increased by 8.1% (Fig. 2A). Similarly, at high TBA concentrations (TBA ≥ 100 µmol/L), more GDM populations were observed (Fig. 2B). Univariate analysis of the ICP with GDM group showed that elevated TBA concentrations were associated with preterm labour (mild 22.6%, moderate 29.2%, severe 54.5%; *p* = 0.034), meconium-stained amniotic fluid (mild 13.4%, moderate 18.5%, severe 45.5%; *p* = 0.01), and low birth weight (mild 24.2%, moderate 35.4%, severe 54.5%; *p* = 0.02) (Supplementary Table 2). In the ICP group, the incidence of PPROM decreased with increasing ICP concentrations (mild 16.6%, moderate 10.3%, severe 7.1%; *p* = 0.013). While the incidence of preterm labour (mild 12.1%, moderate 23.4%, severe 17.9%; *p* < 0.001), meconium-stained amniotic fluid (mild 13.3%%, moderate 17.2%, severe 28.6%; *p* = 0.021), and low birth weight (mild 17.3%, moderate 28.9%, severe 28.6%; *p* = 0.02) were increased with the severity of ICP. Simple logistic regression analysis showed that every doubling of serum TBA concentration increased the risk of all preterm labour by 16.7%, meconium-stained amniotic fluid by 14.2%, and low birth weight by 21.9%. (Fig. 3).

The differences in adverse perinatal outcomes between the ICP with GDM group and the ICP group at different TBA concentrations were further analyzed using logistic regression analysis. In univariate regression analysis, GDM increased the probability of preterm labour and low birth weight in the mild ICP group (Table 3). After adjusting for statistically significant baseline differences as confounders, the risk of preterm labour occurrence remained increased (22.6% relative to 12.1%; OR 1.80; 95% CI 1.30,2.50), however, the incidence of low birth weight (24.2% relative to 17.3%; OR 1.25; 95% CI 0.91,1.71) was not statistically different. There was no significant difference in preterm labour, meconium-stained amniotic fluid, and low birth weight between the ICP with GDM group and the ICP group in moderate ICP and severe ICP populations. Although not statistically significant, the incidence of preterm labour was three times higher in the ICP with GDM group (54.5%) than in the ICP group (17.9%) under the severe ICP population. Women with severe ICP were more likely to have preterm labour (54.5% relative to 17.9%), meconium-stained amniotic fluid (45.5% relative to 28.6%), and low birth weight adverse outcomes (54.5% relative to 28.6%) when they also had GDM.

**Table 3:** The univariate and multivariate analysis of adverse perinatal outcomes with different ICP severity between ICP with GDM group and ICP group

Characteristics	severity of ICP	ICP with GDM (***n*** = 390)	ICP without GDM (***n*** = 1,788)	Univariate analysis		Multivariate analysis*	
				OR (95% CI)	***p***.value	OR (95% CI)	***p***.value
Preterm labour, n, (%)	Mild	71(22.6%)	180(12.1%)	2.12(1.56, 2.88)	< 0.001	1.80(1.30, 2.50)	< 0.001
	Moderate	19(29.2%)	64(23.4%)	1.35(0.74, 2.47)	0.331	1.12(0.59, 2.15)	0.726
	Severe	6(54.5%)	5(17.9%)	5.52(1.19, 25.52)	0.029	9.70(0.95, 99.00)	0.055
Meconium-stained amniotic fluid, n (%)	Mild	42(13.4%)	198(13.3%)	1.00(0.70, 1.44)	0.977	1.02(0.71, 1.48)	0.908
	Moderate	12(18.5%)	47(17.2%)	1.09(0.54, 2.19)	0.812	1.01(0.48, 2.12)	0.987
	Severe	5(45.5%)	8(28.6%)	2.08(0.49, 8.82)	0.319	1.83(0.20, 17.10)	0.595
Low birth weight, n (%)	Mild	76(24.2%)	257(17.3%)	1.53(1.14, 2.05)	0.004	1.25(0.91, 1.71)	0.173
	Moderate	23(35.4%)	79(28.9%)	1.35(0.76, 2.38)	0.310	0.92(0.48, 1.76)	0.795
	Severe	6(54.5%)	8(28.6%)	3.00(0.71, 12.69)	0.136	4.71(0.52, 42.36)	0.167

*Excluded confounders: maternal age, pregestational weight, gestational weight gain, pre-pregnancy body mass index, bile acid concentration, use of ursodeoxycholic acid, ART, and total bile acid

### Construction and verification of preterm labour model in women with ICP

**Fig. 4:**
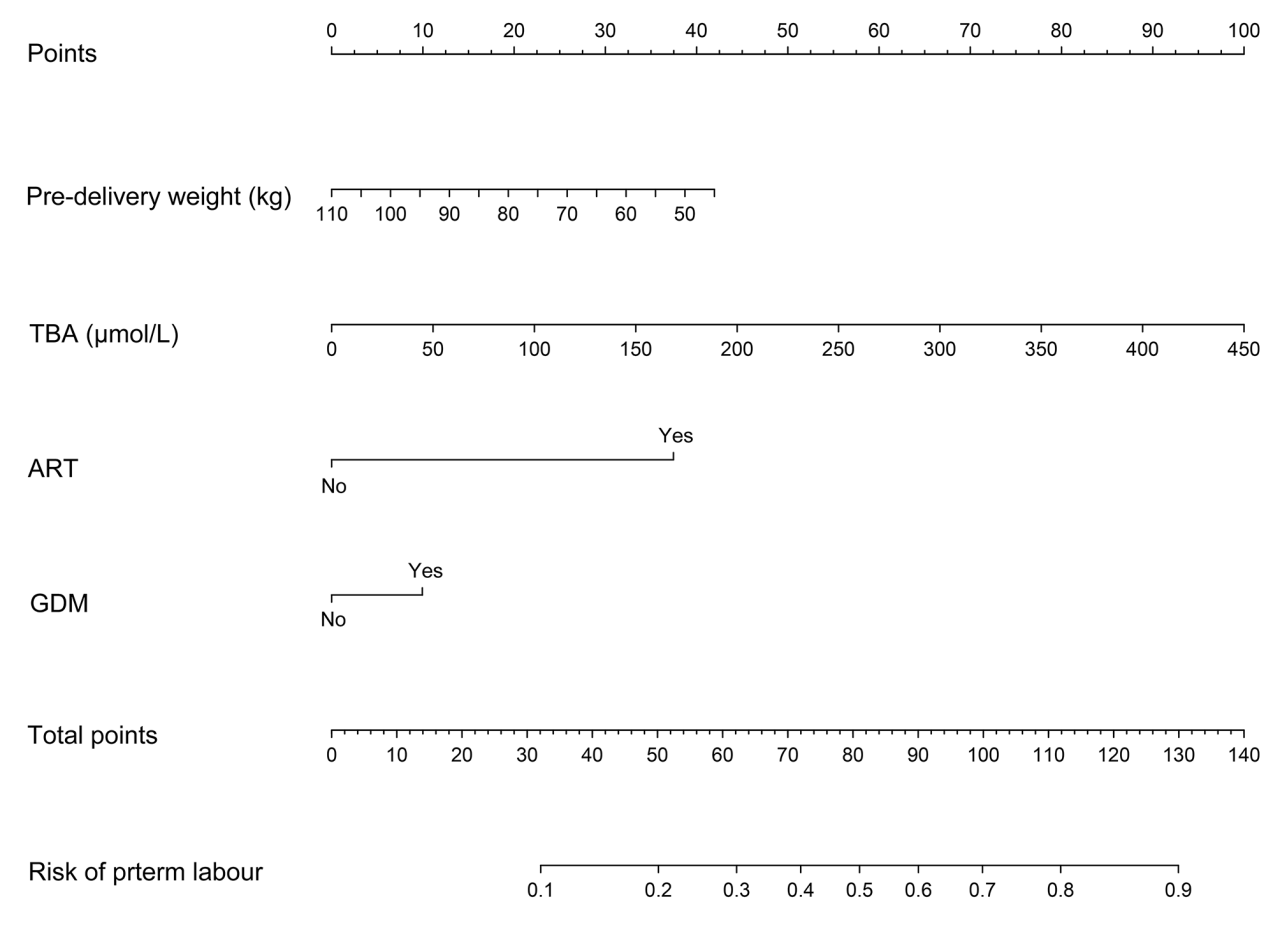
A nomogram prediction model for preterm labour in women with ICP

Considering the high incidence of preterm labour in the ICP population, especially in ICP with GDM, we then constructed a nomogram predictive model of preterm labour. Univariate logistic regression analysis was used to analyze the general clinical data and laboratory serological indicators of 14 variables in all populations. The results showed that five statistically significant variables related to the incidence of preterm labour were selected from 14 variables (*p* < 0.05), including pre-delivery weight, gestational weight gain, TBA, ART, and GDM (Supplementary Table 3). Five variables with significant differences selected by univariate analysis were included in multivariate logistic regression analysis as potential risk factors (Table 4). After adjusting for confounders, four independent risk factors were screened out: pre-delivery weight (OR = 0.97, 95% CI 0.96,0.99), TBA (OR = 1.01, 95% CI 1.00,1.01), ART (OR = 5.10, 95% CI 3.86,6.75), GDM (OR = 1.65, 95% CI 1.23,2.19). The prediction model was developed based on these four independent risk factors and presented in the form of a nomogram (Fig. 4).

**Fig. 5:**
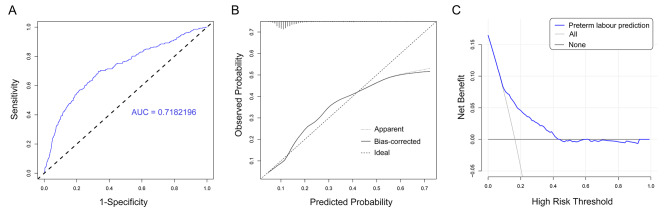
Validation and evaluation of preterm labour prediction model. ROC curve **(A)**, calibration curve **(B)**, and clinical decision curve (DCA) of the training group **(C)**

The ROC curve showed that the AUC value of the training set was 0.718 (Fig. 5A), indicating that the model had a good predictive performance for the risk of preterm labour in the ICP population. The AUC values of the model were recalculated by internal and external validation (test set) to be 0.704 and 0.679 (Supplementary Fig. 1A, 1D). The calibration curve in the training set showed that the model prediction curve was basically consistent with the ideal curve (Fig. 5B), suggesting that the model predicted the risk of preterm labour was consistent with the actual risk, and the model had high accuracy. Compared with the training set, the consistency of the calibration curve of the test set is slightly worse, but the overall shape is generally consistent (Supplementary Fig. 1B, 1E). In addition, to evaluate the clinical practicability of the nomogram, a clinical decision curve was constructed to evaluate the prediction model. The results showed that the blue line was located to the upper right of the all line and none line within a larger threshold probability range (Fig. 5C), indicating the high clinical utility of our constructed nomogram prediction model for the risk of Preterm labour in women with ICP. The clinical decision curve in the validation set was generally consistent with the curve trend in the training set (Supplementary Fig. 1C, 1 F).

**Table 4:** Predictors of preterm labour in women with ICP.

Predictor	Univariate analysis		Multivariate analysis *	
	OR (95% CI)	***p***.value	OR (95% CI)	***p***.value
Pre-delivery weight	0.98(0.97, 1.00)	0.005	0.97(0.96, 0.99)	0.004
Gestational weight gain	0.97(0.95, 1.00)	0.009	0.98(0.95, 1.01)	0.245
Total bile acid	1.01(1.00, 1.01)	0.001	1.01(1.00, 1.01)	0.001
ART	4.87(3.72, 6.37)	< 0.001	5.10(3.86, 6.75)	< 0.001
GDM	2.02(1.55, 2.63)	< 0.001	1.65(1.23, 2.19)	0.001

* Variables with *p* < 0.05 in univariate analysis were excluded as confounders in multivariate analysis

## Discussion

ICP is the most common pregnancy-specific liver disease. Although it poses a high risk to the fetus rather than to pregnant women, it can cause several complications, including complications such as preterm labour, meconium-stained amniotic fluid, fetal distress, and stillbirth [14–16]. These complications were thought to be closely related to increased concentrations of TBA. This study found that elevated TBA concentrations are associated with an increased incidence of adverse pregnancy outcomes, preterm labour, and low birth weight. Previous studies have also indicated a link between maternal TBA concentrations and the risk of adverse pregnancy outcomes [3, 17]. For example, a retrospective cohort study conducted in Sweden found that when TBA ≥ 40 µmol/L, each increase in bile acid concentration by 1–2 µmol/L increased the risk of adverse outcomes by 1–2% [5]. Other studies have subsequently confirmed this relationship. A prospective cohort study in women with severe ICP revealed that the risk of preterm labour, amniotic fluid fecal contamination, and stillbirth increased with the increasing TBA concentrations [18]. A meta-analysis of 5,557 ICP cases and 165,136 controls showed that the risk of stillbirth is increased in women with TBA ≥ 100 µmol/L, and risk of spontaneous Preterm labour is increased in those with TBA ≥ 40 µmol/L [19]. To reduce the incidence of adverse pregnancy outcomes, the Society for Maternal-Fetal Medicine and the Royal College of Obstetricians and Gynaecologists have also developed relevant guidelines, such as delivering the baby in a timely manner based on TBA concentrations between 35 and 40 weeks [20]. Therefore, closely monitoring the TBA concentrations in women with ICP and taking active management measures are beneficial to improve the adverse outcomes.

Currently, several cohort studies have confirmed that pregnant women with ICP are more likely to be with GDM. A 12-year population-based cohort study in Swedish first discovered that pregnant women with ICP had higher rates of gestational diabetes and pre-eclampsia [9]. Subsequently, cohort studies in the United States, Poland, China, and Denmark also reported a higher incidence of GDM in patients with ICP [21–24]. In our study, we found that the incidence of GDM in pregnant women with ICP was as high as 17.91%. However, there are still unresolved issues, such as: the unclear causal relationship between ICP and GDM and the potential impact of ICP with GDM on pregnancy outcomes. Studies have shown that the primary bile acid farnesoid receptor (FXR) was down-regulated or genetic variation in women with ICP, which can further affect glucose homeostasis [25, 26]. Moreover, the incidence of GDM increased significantly with the increase of TBA, ranging from 17.43% in mild ICP to 28.21% in severe ICP, suggesting that ICP may be a cause of GDM. Our study focused on the impact of ICP with GDM on pregnancy outcomes. We found that ICP with GDM led to a higher incidence of preterm labour and polyhydramnios compared to ICP without GDM. It is worth noting that the incidence of preterm labour, meconium-stained amniotic fluid, and low birth weight will significantly increase with the increase of TBA, and ICP with GDM worsened these pregnancy outcomes. We observed that drugs such as ursodeoxycholic acid failed to mitigate the adverse pregnancy outcomes associated with GDM combined with ICP. It is worth noting that TBA concentrations can be influenced by diet. Mitchell et al. have highlighted that for women with moderate ICP (TBA ≥ 40 µmol/l), diagnosis should involve non-fasting samples [27]. They discovered that postprandial TBA concentrations in women with fasting TBA concentrations of < 40 µmol/l could rise to ≥ 40 µmol/l or even higher, reaching ≥ 100 µmol/l. Notably, all the studies included in our analysis relied on fasting TBA concentrations, potentially missing accurate risk stratification. This may explain the absence of discernible differences in perinatal outcomes between the treated and untreated groups. Preterm labour account for more than 15% of deaths among children under 5 years of age, as well as more than half the long-term morbidity [28]. Despite the survival rate of premature infants has been greatly improved with the development of medical nursing, they still susceptible to the threat of neurodevelopmental impairments and respiratory and gastrointestinal complications in the short-term and long-term development [29]. As a result, accurately predicting preterm labour and implementing effective perinatal interventions to prevent associated complications, particularly brain injury and abnormal brain development, is a crucial area of focus for future work [30]. Numerous Preterm labour prediction models for different populations have also been actively established [31–33]. Based on this ICP cohort, we finally developed a nomogram prediction model to predict the incidence of preterm labour. The nomogram prediction model identified TBA concentrations as a significant risk factor for preterm labour, while the contribution of prepartum weight and IVF was also considerable. Although ICP with GDM is a risk factor for preterm labour, it accounts for a small proportion. In general, the data required by our prediction model is easy to obtain, and the prediction accuracy was good. In clinical practice, it can assist doctors in evaluating the likelihood of premature labour of pregnant women with ICP to take intervention measures in advance to reduce the adverse pregnancy outcomes.

### Limitations

Several notable limitations warrant specific emphasis in this study. First, this was a retrospective study, and the design of this study may have been limited by information collection, and there may have been measurement bias in patient self-reported information or data in medical records. Additionally, the temporal relationship between ICP and GDM could not be established in this study. Given the shared physiological and metabolic pathways of GDM and ICP, the absence of a clear temporal relationship makes it challenging to discern their independent contributions to adverse pregnancy outcomes. Future prospective studies are essential to elucidate the temporal dynamics between these two conditions. In addition, it is important to emphasize that this study did not provide information on the treatment of GDM during pregnancy. Treatment regimens have an important impact on the interpretation of study results, and our study failed to comprehensively cover this information. Unknown therapeutic interventions may have an impact on the association between ICP and GDM.

## Conclusion

In conclusion, with the increasing of TBA concentrations there is a corresponding increase in the incidence of GDM, preterm labour, meconium-stained amniotic fluid, and polyhydramnios. When ICP was with GDM, adverse pregnancy outcomes were further exacerbated, especially for pregnant women with severe ICP. Although the nomogram prediction model for preterm labour was effective, there is still room for improvement to achieve the best possible model. Therefore, we plan to expand our cohort and focus on increasing the number of women with severe ICP to optimize the model. Moreover, intervention measures beyond ursodeoxycholic acid should be considered for pregnant women with ICP and GDM.

### Electronic supplementary material

Below is the link to the electronic supplementary material.

Supplementary Material 1

## Data Availability

The data underlying this article will be provided by the corresponding author on reasonable request.
