# Supplementary material for: Gestational diabetes mellitus aggravates adverse perinatal outcomes in women with intrahepatic cholestasis of pregnancy
Source: Diabetol Metab Syndr. 2024 Mar 1;16:57. doi: 10.1186/s13098-024-01294-z (PMC10908036; doi:10.1186/s13098-024-01294-z)
Supplement: Supplementary file 1 — Supplementary Material 1 [file 13098_2024_1294_MOESM1_ESM.docx]

**Gestational diabetes mellitus aggravates adverse perinatal outcomes in women with intrahepatic cholestasis of pregnancy**

Xia Li^a.b.¶^, Qin-Yu Cai^b.c.¶^, Xin Luo^b.d^, Yong-Heng Wang^a.b^, Li-Zhen Shao^a.b^, Shu-Juan Luo^c^, Lan Wang^c^, Ying-Xiong Wang^a.b^, Xia Lan^c.**^, Tai-Hang Liu^a.b.*^

a. Department of Bioinformatics, The School of Basic Medicine, Chongqing Medical University, Chongqing 400016, China.

b. Joint International Research Laboratory of Reproduction & Development, Chongqing Medical University, Chongqing 400016, China.

c. Department of Obstetrics, Women and Children’s Hospital of Chongqing Medical University, Chongqing 401147, China.

d. Department of Obstetrics, The First Affiliated Hospital of Chongqing Medical University, Chongqing 400016, China.

**Correspondence:** Box 197, Chongqing Medical University, No.1 Yixueyuan Rd, Yuzhong District, Chongqing, 400016, PR China. Tel.: +86 023 68485868; Fax: +86 023 68485000. ** Xia Lan (18623177325@163.com); * Tai-Hang Liu (liuth@cqmu.edu.cn)

¶ X. Li and Q. Cai contributed equally to this work.

**Supplementary Table 1. Analysis of the effects of ursodeoxycholic acid on adverse perinatal outcomes in ICP with GDM group.**

| **Variables** | **Use of ursodeoxycholic acid (n=134)** | **Non-use of ursodeoxycholic acid (n=256)** | ***p*.value** |
| --- | --- | --- | --- |
| Preeclampsia, n (%) | 8(6.5%) | 13(5.1%) | .711 |
| Anemia during pregnancy, n (%) | 13(10.5%) | 23(9.0%) | .816 |
| Nuchal cord, n (%) | 31(25.0%) | 80(31.3%) | .092 |
| PPROM, n (%) | 16(12.9%) | 48(18.8%) | .085 |
| Placenta accreta, n (%) | 8(6.5%) | 30(11.7%) | .069 |
| Abnormal placental shape, n (%) | 3(2.4%) | 10(3.9%) | .555 |
| Preterm labour, n (%) | 28(22.6%) | 68(26.6%) | .217 |
| Fetal respiratory distress syndrome, n (%) | 10(8.1%) | 23(9.0%) | .608 |
| Fetal macrosomia, n (%) | 4(3.2%) | 5(2.0%) | .501 |
| Fetal growth restriction, n (%) | 2(1.6%) | 6(2.3%) | .720 |
| NICU admission, n (%) | 23(18.5%) | 43(16.8%) | .927 |
| Meconium-stained amniotic fluid, n (%) | 14(11.3%) | 45(17.6%) | .062 |
| polyhydramnios, n (%) | 3(2.4%) | 15(5.9%) | .106 |
| Oligohydramnios, n (%) | 5(4.0%) | 16(6.3%) | .295 |
| Fetal anomaly, n (%) | 2(1.6%) | 3(1.2%) | .789 |
| Fetal chromosomal abnormalities, n (%) | 0(0.0%) | 1(0.4%) | .469 |
| Low birth weight, n (%) | 42(33.9%) | 63(24.6%) | .154 |

**Abbreviations:** PPROM, preterm prelabor rupture of membranes; NICU, neonatal intensive care unit.

**Supplementary Table 2. Univariate analysis of adverse perinatal outcomes with different ICP severity in ICP with GDM group and ICP group.**

| **Characteristics** | **Subgroups** | **Mild ICP**  **(10≤TBA<40)** | **Moderate ICP**  **(40≤TBA<100)** | **Severe ICP**  **(TBA≥100)** | ***p*.value** |
| --- | --- | --- | --- | --- | --- |
| Preeclampsia, n (%) | ICP with GDM | 18(5.7%) | 3(4.6%) | 0(0.0%) | 1.000 |
|  | ICP without GDM | 87(5.9%) | 20(7.3%) | 3(10.7%) | .388 |
| Anemia during pregnancy, n (%) | ICP with GDM | 26(8.3%) | 9(13.8%) | 1(9.1%) | .369 |
|  | ICP without GDM | 176(11.8%) | 27(9.9%) | 2(7.1%) | .501 |
| Nuchal cord, n (%) | ICP with GDM | 95(30.3%) | 12(18.5%) | 4(36.4%) | .134 |
|  | ICP without GDM | 412(27.7%) | 82(30.0%) | 8(28.6%) | .732 |
| PPROM, n (%) | ICP with GDM | 56(17.8%) | 7(10.8%) | 1(9.1%) | .301 |
|  | ICP without GDM | 247(16.6%) | 28(10.3%) | 2(7.1%) | .013 a |
| Placenta accreta, n (%) | ICP with GDM | 27(8.6%) | 10(15.4%) | 1(9.1%) | .243 |
|  | ICP without GDM | 108(7.3%) | 20(7.3%) | 2(7.1%) | .999 |
| Abnormal placental shape | ICP with GDM | 9(2.9%) | 3(4.6%) | 1(9.1%) | .202 |
|  | ICP without GDM | 43(2.9%) | 6(2.2%) | 0(0.0%) | .858 |
| Preterm labour, n (%) | ICP with GDM | 71(22.6%) | 19(29.2%) | 6(54.5%) | .034 b |
|  | ICP without GDM | 180(12.1%) | 64(23.4%) | 5(17.9%) | <.001 a |
| Fetal respiratory distress syndrome, n (%) | ICP with GDM | 26(8.3%) | 5(7.7%) | 2(7.1%) | .447 |
|  | ICP without GDM | 119(8.0%) | 16(5.9%) | 5(17.9%) | .066 |
| Fetal macrosomia, n (%) | ICP with GDM | 8(2.5%) | 1(1.5%) | 0(0.0%) | 1.000 |
|  | ICP without GDM | 41(2.8%) | 7(2.6%) | 0(0.0%) | 1.000 |
| Fetal growth restriction, n (%) | ICP with GDM | 6(1.9%) | 2(3.1%) | 0(0.0%) | .706 |
|  | ICP without GDM | 26(1.7%) | 5(1.8%) | 1(3.6%) | .468 |
| Neonatal intensive care unit admission, n (%) | ICP with GDM | 49(15.6%) | 13(20.0%) | 4(36.4%) | .151 |
|  | ICP without GDM | 155(10.4%) | 49(17.9%) | 6(21.4%) | .001a |
| Meconium-stained amniotic fluid, n (%) | ICP with GDM | 42(13.4%) | 12(18.5%) | 5(45.5%) | .010 b |
|  | ICP without GDM | 198(13.3%) | 47(17.2%) | 8(28.6%) | .021 |
| Polyhydramnios, n (%) | ICP with GDM | 15(4.8%) | 3(4.6%) | 0(0.0%) | 1.000 |
|  | ICP without GDM | 31(2.1%) | 5(1.8%) | 0(0.0%) | 1.000 |
| Oligohydramnios, n (%) | ICP with GDM | 15(4.8%) | 5(7.7%) | 1(9.1%) | .360 |
|  | ICP without GDM | 150(10.1%) | 30(11.0%) | 0(0.0%) | .183 |
| Fetal anomaly, n (%) | ICP with GDM | 4(1.3%) | 1(1.5%) | 0(0.0%) | 1.000 |
|  | ICP without GDM | 27(1.8%) | 3(1.1%) | 1(3.6%) | .349 |
| Fetal chromosomal abnormalities, n (%) | ICP with GDM | 1(0.3%) | 0(0.0%) | 0(0.0%) | 1.000 |
|  | ICP without GDM | 10(0.7%) | 2(0.7%) | 0(0.0%) | 1.000 |
| Low birth weight, n (%) | ICP with GDM | 76(24.2%) | 23(35.4%) | 6(54.5%) | .020 |
|  | ICP without GDM | 257(17.3%) | 79(28.9%) | 8(28.6%) | <.001 a |

a. Significant difference between Mild group and moderate group.

b. Significant difference between Mild group and Severe group.

**Supplementary Table 3.** **Univariate logistic regression analysis of risk factors for preterm labour in women with ICP.**

| **Characteristics** | **OR (95%CI)** | ***p*.value** |
| --- | --- | --- |
| Maternal age | 1.01(0.98, 1.04) | . 446 |
| Pregestational weight | 0.99(0.97, 1.00) | . 145 |
| pre-delivery weight | 0.98(0.97, 1.00) | . 005 |
| Gestational weight gain | 0.97(0.95, 1.00) | . 009 |
| Pre-pregnancy BMI | 0.99(0.95, 1.03) | . 543 |
| pregnancy BMI | 0.96(0.93, 1.00) | . 053 |
| Total bile acid | 1.01(1.00, 1.01) | . 001 |
| Use of ursodeoxycholic acid | 0.94(0.74, 1.19) | . 577 |
| History of ICP | 0.74(0.45 1.23) | . 245 |
| History of adverse pregnancy | 1.26(0.84, 1.88) | . 268 |
| Smoking | 1.78(0.64, 4.94) | . 266 |
| Drinking | 0.63(0.17, 2.28) | . 478 |
| ART | 4.87(3.72, 6.37) | . 000 |
| GDM | 2.02(1.55, 2.63) | . 000 |

**Abbreviations:** BMl, body mass index; ICP, intrahepatic cholestasis of pregnancy; ART, assisted reproductive technology; GDM, gestational diabetes mellitus.

**Supplementary Figure 1**


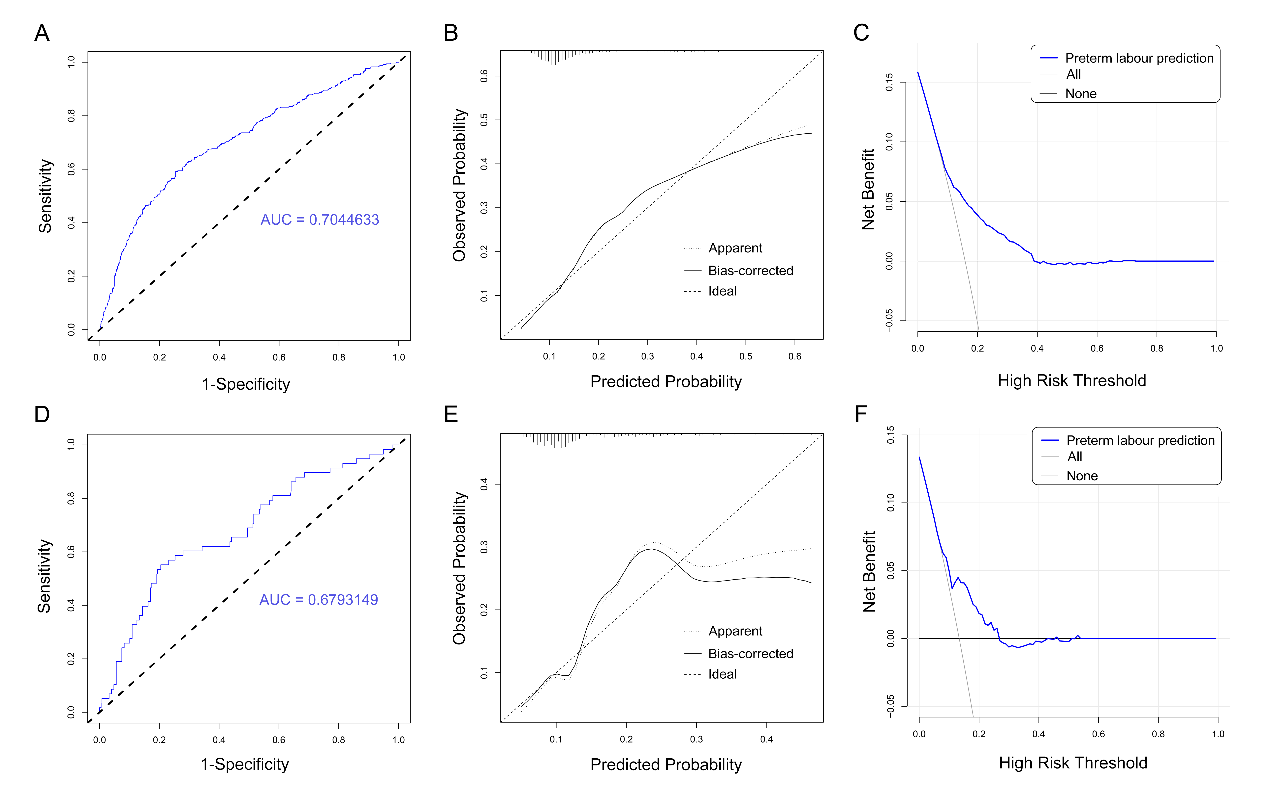


**Validation and evaluation of preterm labour prediction model.** Internally validated ROC curve (A), calibration curve (B), clinical decision curve (C) and externally validated ROC curve (D), calibration curve (E), clinical decision curve (F).
